# Urine Concentrating Capacity, Vasopressin and Copeptin in ADPKD and IgA Nephropathy Patients with Renal Impairment

**DOI:** 10.1371/journal.pone.0169263

**Published:** 2017-01-12

**Authors:** Debbie Zittema, Niek F. Casteleijn, Stephan J. L. Bakker, Lianne S. M. Boesten, A. A. Margreeth Duit, Casper F. M. Franssen, Carlo A. J. M. Gaillard, Ron T. Gansevoort

**Affiliations:** 1 Department of Nephrology, University Medical Center Groningen, University of Groningen, Groningen, The Netherlands; 2 Department of Clinical Chemistry, IJsselland Ziekenhuis, Capelle aan den IJssel, The Netherlands; Universita degli Studi di Bari Aldo Moro, ITALY

## Abstract

**Background:**

Autosomal Dominant Polycystic Kidney Disease (ADPKD) patients have an impaired urine concentrating capacity. Increased circulating vasopressin (AVP) concentrations are supposed to play a role in the progression of ADPKD. We hypothesized that ADPKD patients have a more severely impaired urine concentrating capacity in comparison to other patients with chronic kidney disease at a similar level of kidney function, with consequently an enhanced AVP response to water deprivation with higher circulating AVP concentrations.

**Methods:**

15 ADPKD (eGFR<60) patients and 15 age-, sex- and eGFR-matched controls with IgA nephropathy (IgAN), underwent a water deprivation test to determine maximal urine concentrating capacity. Plasma and urine osmolality, urine aquaporin-2 (AQP2) and plasma AVP and copeptin (a surrogate marker for AVP) were measured at baseline and after water deprivation (average 16 hours). In ADPKD patients, height adjusted total kidney volume (hTKV) was measured by MRI.

**Results:**

Maximal achieved urine concentration was lower in ADPKD compared to IgAN controls (533±138 vs. 642±148 mOsm/kg, p = 0.046), with particularly a lower maximal achieved urine urea concentration (223±74 vs. 299±72 mmol/L, p = 0.008). After water deprivation, plasma osmolality was similar in both groups although change in plasma osmolality was more profound in ADPKD due to a lower baseline plasma osmolality in comparison to IgAN controls. Copeptin and AVP increased significantly in a similar way in both groups. AVP, copeptin and urine AQP2 were inversely associated with maximal urine concentrating in both groups.

**Conclusions:**

ADPKD patients have a more severely impaired maximal urine concentrating capacity with a lower maximal achieved urine urea concentration in comparison to IgAN controls with similar endogenous copeptin and AVP responses.

## Introduction

One of the first clinical features in autosomal dominant polycystic kidney disease (ADPKD) is an impaired urine concentrating capacity that occurs prior to kidney function decline [1–3]. The mechanism leading to decreased urine concentrating capacity is not fully understood. Probably abnormalities in the renal medullary architecture, due to cyst formation and expansion, play an important role. In a previous study, we found that already in the early stages of disease there is an impaired maximal urine concentrating capacity, which is accompanied by increased plasma osmolality and vasopressin (AVP) levels during water deprivation, in comparison to healthy controls [4].

AVP is secreted from the pituitary gland when plasma osmolality increases. AVP subsequently binds to the vasopressin V2 receptor of the collecting ducts which stimulates water reabsorption by migration of aquaporin-2 (AQP2) to the apical cell membrane. Besides being important for water homeostasis, AVP has deleterious effects in ADPKD. AVP has been shown to increase intracellular cAMP, which promotes cell proliferation and cyst formation [5]. Indeed, animal models and a large randomized controlled trial in ADPKD patients showed that blocking the vasopressin V2 receptor reduces the rate of cyst growth and renal function loss [6–9].

In the present study, we hypothesized that in advanced stages of ADPKD, the increase in AVP in response to water deprivation is stronger than might be expected from impaired kidney function per se [10,11]. To study urine concentrating capacity and AVP response in ADPKD, we performed water deprivation tests in ADPKD patients with impaired kidney function and in a control group of patients with IgA nephropathy (IgAN), matched for age, sex and eGFR. In addition to AVP, copeptin was measured as a surrogate marker for AVP, since copeptin is more stable than AVP [12–14].

## Subjects and Methods

### Study population

Eligible for this study were patients with ADPKD, as diagnosed using the revised Ravine criteria [15], aged between 18–65 years and with an estimated GFR (eGFR) <60 ml/min/1.73m^2^. The control group consisted of IgA nephropathy (IgAN) patients, matched for eGFR, age and sex. The diagnosis of IgAN was based on renal biopsy or clinical history and laboratory values in accordance with clinical practice. IgAN patients were eligible when they were in a stable phase of their disease, as defined by proteinuria <1 g/d and eGFR loss ≤5 ml/min/1.73m^2^ in the previous year and without use of immunosuppressive medication. Exclusion criteria for both patient groups were: use of medications or concomitant diseases that influence urine concentration capacity other than ADPKD or IgAN (e.g., diuretics, lithium and diabetes mellitus), factors that may influence urine concentration capacity (e.g. smoking, menstruation, urinary tract infection, pregnancy, and consumption of ≥4 units of alcohol per day) or active cardiovascular disease (e.g. angina pectoris), which is a contraindication for DDAVP administration. This study was approved by our institutional review board, the Medical Ethical Committee of the University Medical Center Groningen, and was performed in adherence to the Declaration of Helsinki. All participants gave written informed consent.

### Study protocol

All patients routinely collected a 24-hour urine sample the day preceding the water deprivation test. Patients underwent a standard prolonged water deprivation test, based on the protocol originally described by Miller *et al*. [16]. The day before the water deprivation test and during the test, participants were not allowed to smoke, drink alcohol or consume caffeine-containing products. At the day of the test, a baseline spot urine sample was collected at 5 p.m. and blood was drawn for direct biochemical evaluation. Plasma was separated and stored at -80°C for later assessment of copeptin and AVP. Thereafter, participants received a standardized meal and were not allowed to eat or drink anymore until the end of the water deprivation test. Patients spent the evening and the night at home. The following day patients returned to the hospital at 8 a.m., after 14 hours of thirsting. Patients spent the day in the hospital, with spot urine samples being collected every hour until two consecutive measurements showed an increase in urine osmolality ≤30 mOsm/kg. After reaching this plateau, participants received an intramuscular injection of 2 mcg DDAVP, a synthetic replacement for AVP. Two hours after injection, blood and urine samples were collected. Urine osmolality that was measured at this time point was used to define maximal urine concentrating capacity. Two hours after injection of DDAVP, participants were allowed to drink and eat ad libitum. To ensure patient safety during the water deprivation test stopping criteria were defined as reaching a body weight reduction >3% or a plasma sodium >150 mmol/L.

### Measurements

Standard biochemical evaluation was performed in fresh urine and plasma samples, using a Roche Modular Autoanalyser (Hitachi, Tokyo, Japan). Plasma and urine osmolality were measured directly via determination of freezing point depression using an Osmometer (Arkray, Kyoto, Japan), with an intra-assay coefficient of variation <1.0%. eGFR was calculated with the CKD-EPI (Chronic Kidney Disease Epidemiology Collaboration) equation [17].

Blood for AVP and copeptin measurement was drawn into a chilled EDTA tube, and immediately centrifuged at 4°C and stored at −80°C until assay. AVP was measured by RIA after an extraction using ODS-silica (DiaSorin, Stillwater, MN). The lower limit of detection was 0.2 pg/ml and the intra-assay coefficient of variation 3.5%. Copeptin was measured using a sandwich immunoassay (B.R.A.H.M.S. AG, Hennigsdorf/Berlin, Germany), with a lower limit of detection of 0.4 pmol/L and intra-assay coefficient of variation of 4 and 3% for the copeptin concentrations of 15 and 50 pmol/L, respectively. Urine aquaporin-2 (AQP2) concentration was measured by a direct ELISA [18] using rabbit-anti-AQP2 antibody (Santa Cruz Biotechnology, Dallas, TX, USA) with a lower limit of detection of 6.67 ng/mL and intra-assay coefficient of variation of 6.1%. In all ADPKD patients MR imaging was performed, using a standardised abdominal MR imaging protocol without the use of intravenous contrast [19]. Total kidney volume (TKV) was assessed using Analyze Direct 8.0 software (AnalyzeDirect, Inc., Overland Park, KS, USA). Total kidney volume was divided by height to calculate the height adjusted total kidney volume (hTKV).

### Statistical analyses

Normally distributed variables are expressed as mean±SD, non-normally distributed variables as median (IQR). Differences in baseline characteristics between ADPKD and IgAN patients were calculated with a Chi-square test for categorical data, and for continuous data with a Student’s t-test or a Mann-Whitney U test in case of non-normally distributed data. Percentage change between baseline and maximal urine concentration were tested in the overall population and within study groups using a one sample t-test with 0% change as reference value. Linear regression analyses were performed to test associations between plasma and urine osmolality, AVP, copeptin, AQP2-creatinine ratio, hTKV, albumin-creatinine ratio (ACR) and urine-to-plasma urea ratio (U/P Urea). AVP, copeptin, AQP2-creatinine ratio, hTKV, ACR and U/P Urea were log (ln) transformed to fulfill the requirement of normal distribution of the residuals for regression analysis. To investigate differences between the two study groups the categorical variable ‘study group’ (ADPKD vs. IgAN) was added to the regression analysis. Furthermore, to investigate whether associations between copeptin and other study variables were different between the study groups, interaction was tested by adding product terms including ‘study group’ and the independent variable to this model. Univariate (crude) linear regression models are presented with the correlation coefficient whereas for multiple variable models the standardized regression coefficient beta (St. β) is given.

All statistical analyses were performed using SPSS 22 (SPSS Statistics, Inc., Chicago, IL, U.S.A.). A p-value of <0.05 was considered to indicate statistical significance and all statistical tests were 2-tailed.

## Results

### Before water deprivation at baseline

Baseline characteristics with respect to age, sex and eGFR were similar between ADPKD patients and the IgAN controls, indicating that matching was successful (Table 1). Blood pressure was slightly higher and 24-hour urine volume was particularly higher in ADPKD patients than in IgAN controls. Total solute, urea and creatinine excretion did not differ between the groups, indicating that both groups had similar nutritional intake and muscle mass. Baseline plasma osmolality, copeptin and AVP were similar in both study groups, although plasma osmolality tended to be lower in ADPKD patients than in IgAN controls (Table 2). A spot urine sample collected before start of the water deprivation test, showed less concentrated urine with lower urine osmolality, sodium and urea in ADPKD patients than IgAN controls (Table 3).

**Table 1:** Characteristics of the overall population, and of ADPKD and IgA Nephropathy patients separately.

	Overall n = 30	ADPKD n = 15	IgAN n = 15	P-value
Age (y)	49±8	49±7	49±9	0.91
Male (%)	66.7	66.7	66.7	1.00
BMI (kg/m^2^)	28±4	27±3	29±4	0.10
BSA (m^2^)	2.06±0.20	2.00±0.19	2.13±0.18	0.06
Systolic blood pressure (mmHg)	129±13	134±14	123±8	0.02
Diastolic blood pressure (mmHg)	81±9	85±10	78±6	0.03
Using antihypertensives (%)	93.3	93.3	93.3	1.00
eGFR (mL/min/1.73m^2^)	47±14	46±11	48±17	0.38
TKV (L)		1.7 (0.9–2.5)		
hTKV (L/m)		1.0 (0.5–1.3)		
***24-hour urine***				
Volume (L)	2.3±0.9	2.8±0.9	1.9±0.5	0.002
Osmolality (mOsmol/kg)	422±144	347±133	496±117	0.003
Osmolality excretion (mOsmol/24h)	431±99	441±88	421±111	0.59
Urea (mmol/L)	190±69	146±53	233±55	<0.001
Urea excretion (mmol/24h)	194±54	201±64	187±43	0.50
Creatinine (mmol/L)	6.7±2.8	5.1±1.9	8.4±2.6	0.001
Creatinine excretion (mmol/24h)	6.7±1.3	6.5±1.2	6.9±1.5	0.39
Albumin excretion (mg/24h)	95 (25–360)	47 (16–288)	148 (68–522)	0.045
Albumin/creatinine ratio (mg/mmol)	9 (2–21)	3 (1–19)	11 (6–39)	0.06
AQP2 excretion (ng/24h)	760 (549–2280)	752 (418–2239)	760 (605–3185)	0.62
AQP2/creatinine ratio (μg/mmol)	62 (45–204)	69 (32–206)	58 (45–204)	1.00

ADPKD and IgA Nephropathy patients were matched for age, sex, and eGFR. Data are given as mean±SD for normally distributed data or median (IQR) for non-normally distributed data. Significance was tested using a chi-square test for categorical data, Student’s t-test for normally distributed data or a Mann-Whitney U test for non-normally distributed data. Osmolality, urea and creatinine excretion were adjusted for BSA. Abbreviations: IgAN, IgA Nephropathy; BMI, body mass index; BSA, body surface area; eGFR, estimated glomerular filtration rate; TKV, total kidney volume; hTKV, height adjusted total kidney volume; AQP2, aquaporin-2.

**Table 2:** Measurements in plasma at baseline (5 p.m.) and at maximal urine concentration during a standard prolonged water deprivation test.

Plasma	Overall n = 30	ADPKD n = 15	IgAN n = 15	P-value
***At baseline***				
Osmolality (mOsmol/kg)	291±8	289±5	294±10	0.14
Sodium (mmol/L)	140±2.5	141±2.9	140±2.0	0.67
Potassium (mmol/L)	4.3±0.4	4.3±0.4	4.3±0.5	0.97
Urea (mmol/L)	11.2±5.3	10.3±3.7	12.1±6.6	0.35
AVP (pmol/L)	4.4 (1.4–12.0)	2.2 (1.3–14.0)	6.3 (1.4–12.0)	0.49
Copeptin (pmol/L)	11.9 (7.1–28.3)	14.0 (6.1–30.1)	11.9 (7.3–27.7)	0.98
***At maximal urine concentration***				
Osmolality (mOsmol/kg)	294±8	293±6	295±10	0.51
Sodium (mmol/L)	142±1.9	142±2.4	141±1.0	0.10
Potassium (mmol/L)	4.4±0.5	4.3±0.5	4.5±0.6	0.30
Urea (mmol/L)	11.0±5.3	10.3±3.5	11.7±6.6	0.47
AVP (pmol/L)	9.6 (2.4–12.3)	9.2 (1.4–12.0)	10.0 (2.5–13.0)	0.57
Copeptin (pmol/L)	23.7 (10.6–44.6)	26.6 (12.7–43.0)	20.7 (10.0–48.3)	0.84
***Change between baseline and maximal urine concentration***				
Osmolality (%)	0.8±1.2*	1.1±1.2*	0.5±1.1	0.09
Sodium (%)	0.8±1.3*	1.0±1.5*	0.7±1.1*	0.32
Potassium (%)	2.2±6.7	0.1±6.5	4.2±6.5*	0.08
Urea (%)	-3.2±11.6	-0.2±7.4	-6.0±14.1	0.14
AVP (%)	86±158*	116±208*	35±61*	0.11
Copeptin (%)	82±89*	94±113*	72±59*	0.51

Data are given as mean±SD for normally distributed data or as median (IQR) for non-normally distributed data. Significance between groups was tested using Student’s t-test for normally distributed data or a Mann-Whitney U test for non-normally distributed data. Percentage change within groups was tested using a one-sample t-test,

* p<0.05.

Abbreviations: IgAN, IgA Nephropathy; AVP, vasopressin; AQP2, aquaporin-2.

**Table 3:** Measurements in spot urine at baseline (5 p.m.) and at maximal urine concentration during a standard prolonged water deprivation test.

Spot urine	Overall n = 30	ADPKD n = 15	IgAN n = 15	P-value
***At baseline***				
Osmolality (mOsmol/kg)	438±160	378±157	498±144	0.04
Sodium (mmol/L)	66±32	55±29	77±32	0.06
Potassium (mmol/L)	46±21	44±20	49±23	0.46
Urea (mmol/L)	207±81	177±80	237±71	0.04
Creatinine (mmol/L)	7.9±3.8	7.2±4.3	8.7±3.3	0.28
Albumin (mg/L)	98 (38–218)	64 (21–127)	137 (64–476)	0.045
Albumin/creatinine ratio (mg/mmol)	14 (7–29)	9 (3–26)	19 (9–51)	0.10
AQP2 (ng/mL)	986 (283–3396)	584 (171–3297)	1562 (293–3693)	0.54
AQP2/creatinine ratio (μg/mmol)	177 (45–361)	106 (28–532)	189 (50–325)	0.87
***At maximal urine concentration***				
Osmolality (mOsm/kg)	587±151	533±138	642±148	0.046
Sodium (mmol/L)	77±31	75±24	80±38	0.65
Potassium (mmol/L)	81±35	78±28	84±41	0.61
Urea (mmol/L)	261±81	223±74	299±72	0.008
Creatinine (mmol/L)	12.8±4.7	11.5±4.2	14.1±5.1	0.14
Albumin (mg/L)	92 (57–245)	64 (26–130)	160 (86–554)	0.01
Albumin/creatinine ratio (mg/mmol)	10 (4–49)	7 (-13)	11 (9–6)	0.045
AQP2 (ng/mL)	676 (343–1444)	410 (274–2844)	833 (425–1274)	0.33
AQP/creatinine ratio (μg/mmol)	52 (31–130)	33 (21–290)	56 (39–109)	0.60
***Change between baseline and maximal urine concentration***				
Osmolality (%)	52±81*	68±107*	36±41*	0.29
Sodium (%)	37±83*	61±93*	12±66	0.11
Potassium (%)	126±266*	171±370	82±73*	0.37
Urea (%)	40±59*	48±73*	33±42*	0.13
Creatinine (%)	98±139*	118±184*	78±75*	0.44
Albumin (%)	122±455	198±641	45±69*	0.09
Albumin/creatinine ratio (%)	-10±60	-7±73	-12±47	0.41
AQP2 (%)	8±98	20±122	-4±68	0.32
AQP/creatinine ratio (%)	-43±44*	-48±22*	-37±59*	0.08

Data are given as mean±SD for normally distributed data or as median (IQR) for non-normally distributed data. Significance was tested using Student’s t-test for normally distributed data or a Mann-Whitney U test for non-normally distributed data. Percentage change within groups was tested using a one-sample t-test,

* p<0.05.

Abbreviations: IgAN, IgA Nephropathy; AVP, vasopressin; AQP2, aquaporin-2.

### After water deprivation at maximal urine concentration

All patients underwent a standard prolonged water deprivation test. None of the patients met the safety stopping criteria during the test. Plasma osmolality increased significantly in ADPKD patients but not in IgAN controls. Upon water deprivation, copeptin and AVP increased significantly in ADPKD patients and IgAN controls in a similar way (Table 2). Urine osmolality increased both in ADPKD patients and IgAN controls (Table 3). However, the maximal achieved urine osmolality was significantly lower in ADPKD patients in comparison to IgAN controls, especially due to a decreased urine urea concentration. AQP2 at maximal urine concentration was similar in both groups and decreased in a similar way during water deprivation. After DDAVP administration, urine osmolality increased in ADPKD patients with an average of +4.7% (p = 0.001). However, numerically this increase was small and similar to the increase in the IgAN control group (+4.5%, p = 0.01, ADPKD vs. IgAN: p = 0.4).

### Associations between copeptin, AVP, plasma and urine osmolality and AQP2

**Fig 1:**
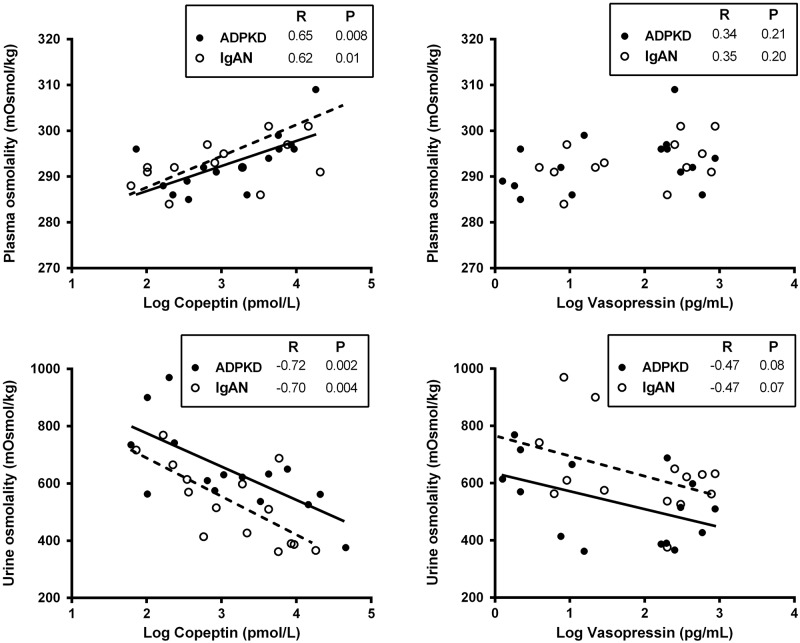
Associations of copeptin and vasopressin concentration with plasma osmolality and urine osmolality in ADPKD patients (solid line) and IgA Nephropathy patients (IgAN, dashed line) at maximal urine concentration.

At baseline and at maximal urine concentration, copeptin and AVP concentrations were strongly associated (R = 0.72 and R = 0.78, respectively, both p<0.001). Furthermore, copeptin was associated with plasma osmolality, a stimulus for AVP release, both at baseline and at maximal urine concentration Table 4 and Fig 1). No interactions by study group for the associations between copeptin and plasma osmolality were found (Table 4). The aforementioned associations were also tested for AVP instead of copeptin, which rendered essentially similar results, albeit that the associations were less strong (Table 4).

**Table 4:** Univariate linear regression associations of plasma copeptin and AVP (log transformed) with plasma osmolality and multivariable linear regression analyses testing the effect of having ADPKD on the associations at baseline and at maximal urine concentration.

*Plasma copeptin*	Crude		Model 1		Model 2	
	R	P-value	St. β	P-value	St. β	P-value
***Baseline***						
Plasma osmolality	0.60	0.001	0.64	<0.001	0.57	0.004
Study group (ADPKD vs. IgAN)			0.14	0.40	-5.3	0.46
Plasma osmolality x Study group					5.4	0.45
***Maximal urine concentration***						
Plasma osmolality	0.62	<0.001	0.63	<0.001	0.57	0.004
Study group (ADPKD vs. IgAN)			0.10	0.51	-3.8	0.54
Plasma osmolality x Study group					3.9	0.53
***Plasma AVP***						
***Baseline***						
Plasma osmolality	0.32	0.09	0.29	0.14	0.26	0.25
Study group (ADPKD vs. IgAN)			-0.10	0.61	-2.77	0.74
Plasma osmolality x Study group					2.66	0.75
***Maximal urine concentration***						
Plasma osmolality	0.34	0.06	0.34	0.08	0.29	0.20
Study group (ADPKD vs. IgAN)			-0.03	0.85	-3.35	0.66
Plasma osmolality x Study group					3.31	0.66

Standardized betas (St. β) and p-values were calculated using multivariable linear regression. Dependent variables are plasma copeptin and AVP (log transformed), independent variables are plasma osmolality, the categorical variable study group (1 = ADPKD, 0 = IgAN) and the interaction term between plasma osmolality and study group. Abbreviations: IgAN, IgA nepghropathy; AVP, vasopressin.

Urine osmolality was inversely associated with copeptin at maximal urine concentration (Fig 1). Addition of the categorical variable study group to the linear regression model, with maximal urine osmolality as dependent variable, showed that ADPKD patients had a 105 mOsmol/kg lower maximal urine osmolality compared with the control group at a similar copeptin level (St. β = -0.35, p = 0.01, Table 5). No interactions by study group for the association between copeptin and urine osmolality was found. AVP was associated with maximal urine osmolality in a similar way, with a 119 mOsmol/kg lower maximal urine osmolality in ADPKD patients at a similar AVP level (St. β = -0.40, p = 0.02, Table 5).

**Table 5:** Linear regression analyses of urine osmolality with plasma copeptin, AVP and urine AQP2/creatinine ratio (all log transformed) at maximal urine concentration, including analyses testing whether study group (i.e. having ADPKD) interacts with these associations.

*Urine osmolality*	Crude		Model 1		Model 2	
	R	P-value	St. β	P-value	St. β	P-value
***Plasma copeptin***						
Plasma copeptin	-0.66	<0.001	-0.66	<0.001	-0.61	0.001
Study group (ADPKD vs. IgAN)			-0.35	0.01	-0.13	0.81
Plasma copeptin x Study group					-0.24	0.67
***Plasma AVP***						
Plasma AVP	-0.41	0.03	-0.44	0.01	-0.47	0.06
Study group (ADPKD vs. IgAN)			-0.40	0.02	-0.45	0.17
Plasma AVP x Study group					0.06	0.87
***Urine AQP2/creatinine***						
Urine AQP2/creatinine	-0.51	0.004	-0.52	0.002	-0.58	0.06
Study group (ADPKD vs. IgAN)			-0.37	0.02	-0.53	0.44
Urine AQP2/creatinine x Study group					0.17	0.82

Standardized beta coefficients (St. β) and p-values were calculated using linear regression. Dependent variable is urine osmolality, independent variables are plasma copeptin (log transformed), plasma AVP (log transformed), urine AQP2/creatinine (log transformed), the categorical variable study group and the interaction term between plasma copeptin, AVP or urine AQP2/creatinine and study group. Abbreviations: IgAN, IgA Nephropathy; AVP, vasopressin; AQP2, aquaporin-2.

The AQP2 to creatinine ratio at maximal urine concentration was inversely associated with maximal urine osmolality. ADPKD patients had a 110 mOsmol/kg lower maximal urine osmolality in comparison with the control group at a similar AQP2 level (St. β = -0.37, p = 0.02, Table 5). No interactions by study group for the association between AQP2 and the maximal urine concentrating capacity was found. Furthermore, AQP2 at maximal urine concentration was positively associated with both copeptin and AVP (Table 6). Having ADPKD or IgAN did not affect these associations (i.e., no significant interactions with study group).

**Table 6:** Univariate linear regression associations of the urine AQP2 to creatinine ratio with plasma copeptin or AVP (both log transformed) at maximal urine concentration and multivariable linear regression analyses testing the effect of having ADPKD on these associations.

*Urine AQP2/creatinine*	Crude		Model 1		Model 2	
	R	P-value	St. β	P-value	St. β	P-value
***Plasma copeptin***						
Plasma copeptin	0.49	0.006	0.49	0.006	0.38	0.09
Study group (ADPKD vs. IgAN)			-0.02	0.92	-0.59	0.40
Plasma copeptin x Study group					0.61	0.40
***Plasma AVP***						
Plasma AVP	0.45	0.01	0.46	0.01	0.33	0.20
Study group (ADPKD vs. IgAN)			0.03	0.87	-0.17	0.63
Plasma AVP x Study group					0.25	0.52

Standardized beta coefficients (St. β) and p-values were calculated using multivariable linear regression. Dependent variable is urine AQP2/creatinine (log transformed), independent variables are plasma copeptin (log transformed), plasma AVP (log transformed), the categorical variable study group (1 = ADPKD, 0 = IgAN) and the interaction term between plasma copeptin or AVP and study group. Abbreviations: AQP2, aquaporin-2; IgAN, IgA Nephropathy; AVP, vasopressin.

### Associations between copeptin and kidney damage

**Fig 2:**
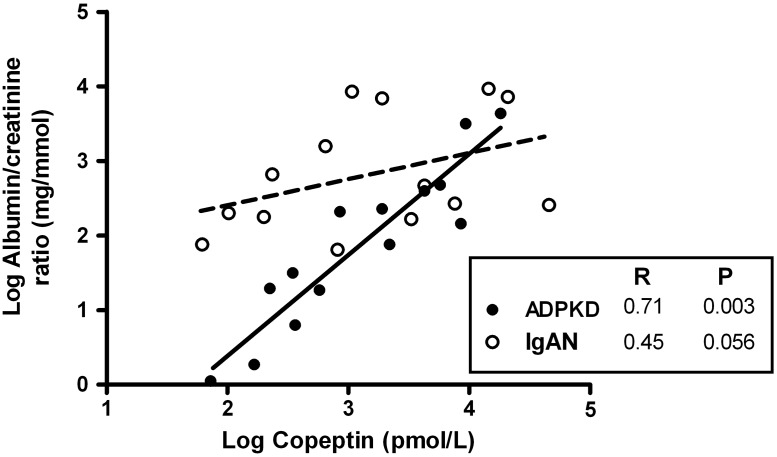
Associations of plasma copeptin concentration with urine albumin to creatinine ratio in ADPKD (solid line) and IgA Nephropathy (IgAN, dashed line) patients at maximal urine concentration.

We investigated whether copeptin was associated with kidney damage. In ADPKD, copeptin at baseline was univariately associated with the urine albumin to creatine ratio (ACR) (R = 0.88, p<0.001) and this held also true at maximal urine concentration (R = 0.71, p = 0.003, Fig 2). The association remained significant after multivariable adjustment for eGFR and hTKV at baseline (St. β = 0.82, p = 0.001) and was of borderline significance at maximal urine concentration (St. β = 0.58, p = 0.06). In the IgAN control group copeptin was not associated with ACR at baseline, neither crude (p = 0.2) nor after adjustment for eGFR (p = 0.7). At maximal urine concentration copeptin tended to be associated with ACR in IgAN controls (R = 0.50, p = 0.06), but this association lost significance after adjustment for eGFR (p = 0.4). In ADPKD, copeptin was furthermore associated with hTKV (R = 0.58, p = 0.03). Of note, hTKV was positively associated with plasma osmolality and inversely with urine osmolality at maximal urine concentration (R = 0.52, p = 0.048, R = -0.54, p = 0.04, respectively).

### Baseline U/P Urea as marker for maximal urine concentration capacity

**Fig 3:**
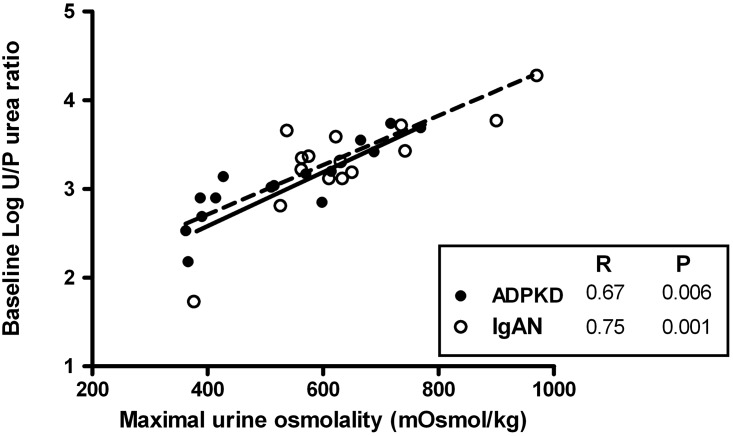
Associations of maximal urine osmolality with baseline urine-to-plasma (U/P) urea ratio in ADPKD patients (solid line) and IgA Nephropathy patients (IgAN, dashed line).

In a previous study, we suggested that the urine-to-plasma urea ratio (U/P Urea), measured routinely in an out-patient clinic setting, may be a marker for maximal urine concentrating capacity [20]. We therefore tested in this study also the association between baseline U/P Urea ratio and maximal urine osmolality. In the two study groups combined (R = 0.73, p<0.001), as well as in both groups separately, strong associations were found (ADPKD: R = 0.67, p = 0.006; IgAN control: R = 0.75, p = 0.001, Fig 3). In the total study group, the association remained significant after adjustment for age, sex and eGFR (St. β = 0.62, p = 0.003) and showed a trend towards significance in the separate study groups (ADPKD: St. β = 0.51, p = 0.1 and IgAN control: St. β = 0.76, p = 0.054). In addition, we tested whether U/P Urea is a marker for disease severity in ADPKD. Significant associations were found for baseline U/P urea with hTKV (R = -0.53, p = 0.04) and eGFR (R = 0.60, p = 0.02), and with copeptin at maximal urine concentration (R = -0.58, p = 0.03).

## Discussion

In the present study we found a more severely impaired urine concentrating capacity in ADPKD patients with, surprisingly, similar AVP and copeptin responses in comparison with IgAN control patients at similar low kidney function. Furthermore, more severe ADPKD, assessed as a higher total kidney volume, was positively associated with plasma osmolality, copeptin and albuminuria, and with a more severely impaired urine concentrating capacity during water deprivation.

After water deprivation, concentrations of plasma AVP, plasma copeptin and urine AQP2 were similar in both study groups, whereas the maximal urine concentrating capacity was significantly more impaired in ADPKD patients. This shows that the process of urine concentration is complex and comprises more than solely variation in the permeability of collecting duct cells. In addition the medullary osmotic gradient is of great importance. This gradient is determined by a complex mechanism involving intra-renal urea recycling by urea transporters in the renal medulla. The importance of these urea transporters for urine concentration has been confirmed in knock-out mouse models [21,22]. Mice with a defect in one or multiple urea transporters were still able to concentrate urine, but to a lesser extent than wild-type mice, due to a reduced urea clearance, whereas sodium and other electrolytes were cleared in a similar way. In APDKD signs of such a urea selective concentrating defect can be observed as well. In a previous study we found that ADPKD patients with preserved kidney function had at maximal urine concentration markedly lower urine urea levels compared to healthy controls (280±56 mmol/L vs. 405±110 mmol/L, p = 0.001) [4]. We hypothesized that in ADPKD patients cyst formation disrupts the medullar osmotic gradient and urea recycling. The present study suggests that this difference in solute clearance seems ADPKD-specific and is not part of kidney damage in general, as urine urea levels at maximal urine concentration were lower in ADPKD patients than in the IgAN control group despite similar level of impaired kidney function. In addition, when comparing urine urea concentrations at the moment of maximal urine concentration in ADPKD with preserved kidney function [23] with findings from the present study, it shows that urine urea concentration decreases when disease progresses (preserved kidney function: 280±56 mmol/L, impaired kidney function: 223±74 mmol/L, p = 0.03). The fact that the U/P urea ratio correlates well with maximal urine concentrating capacity shows the importance of urea in the urine concentration process as well.

After water deprivation, the increase in copeptin and AVP was similar in both study groups, even though the maximal urine concentrating capacity was more impaired and the increase in plasma osmolality seemed more profound in ADPKD patients in comparison to the control group. In both groups plasma osmolality was comparable at the end of the water deprivation test, which could be the explanation for similar copeptin and AVP levels at the moment of maximal urine concentration. On the other hand, Ho et al. have described the possibility of a central component causing the impaired urine concentrating capacity in ADPKD. These authors hypothesized that expression of *PKD1* and *PKD2* transcripts in hypothalamic nuclei that synthesize AVP could be involved [24]. They found in ADPKD patients a lower maximal urine osmolality in comparison with healthy controls, but no AVP response during water deprivation. They also did not find an association between AVP and plasma osmolality, and suggested that AVP secretion was blunted in ADPKD patients. In our study, a significant response in both copeptin and AVP was seen. Nevertheless, we found no association between plasma osmolality and AVP as well, suggesting that a central component may play a role. However, an association between plasma osmolality and copeptin was present. The latter suggests that copeptin and therefore also AVP secretion responded appropriately to plasma osmolality, which makes a central component less likely. The contradictory results between AVP and copeptin that are seen in our study may be explained by differences in assay sensitivity, as copeptin is more stable than AVP ex-vivo and therefore probably more reliable to measure [4,12–14]. Based on our results a central component in the impaired maximal urine concentration capacity in ADPKD seems unlikely but cannot be excluded.

In this study ADPKD patients with later stages of disease showed markedly higher AVP and copeptin levels at the end of a water deprivation test compared to levels that were achieved in our previous study that was performed in ADPKD patients with earlier stages of disease (9.2 (1.4–12.0) pmol/L vs. 1.6 (1.13–2.41) pmol/L, p = 0.007) [4]. It is assumed that AVP has a detrimental role in ADPKD, because it leads to an increase in intracellular cAMP in distal tubular cells, which in turn leads to cell proliferation and increased fluid section, the processes that drive cyst formation and growth [25]. When cysts are formed and expand because of a genetic defect, urine concentrating capacity decreases, leading to an increase in AVP and consequently to even more cyst formation and expansion. Thus a vicious circle is created that predisposes for kidney growth and loss of kidney function. To reduce cyst growth, an increase in AVP levels should be avoided. Our study results indicate that thirsting enhances AVP release, also in ADPKD, and suggest that dehydration should be avoided in this patient group.

The major strength of our study is the inclusion of a control group of eGFR-, age- and sex-matched IgAN patients. This allowed us to conclude whether our observations in ADPKD patients are disease specific or due to impaired eGFR, without misinterpreting data due to differences in age and sex distribution. These latter factors have been shown to be associated with maximal urine concentrating capacity [11,26,27]. In addition, we measured both AVP and copeptin levels. Therefore we were able to confirm outcomes with respect to AVP that showed a trend toward significance, with copeptin values that are more easy and reliable to measure. Using copeptin levels we indeed were able to detect more subtle associations and differences between the two study groups. Some limitations need to be addressed. First, the relatively small sample size. No data on urine concentration capacity of IgAN patients was available from literature to perform a power calculation a priori. Therefore the size of our study population was based on experience obtained in a previous water deprivation test [4]. Although differences between the present study groups were less profound compared to the differences between study groups in our previous study, our main findings are clear and well powered (i.e., statistically significant). Second, 24h urine volume was significantly higher in ADPKD patients in comparison to IgAN controls. This was probably due to the fact that ADPKD patients in our department are used to hydrate properly. Because baseline was not standardized, this led to lower urine and plasma osmolality in ADPKD patients at baseline. However, our conclusions are based on the standardized results found after water deprivation. Third, our study design may not be optimal to detect a central component causing partial diabetes insipidus. We used a standard prolonged water deprivation test, which can distinguish between a complete central or nephrogenic origin of diabetes insipidus, but is less accurate in detecting partial and especially mixed syndromes [16]. Lastly, the control group consisted of only IgAN patients. We preferred this option over including patients with a case mix of diseases with uncertain results.

In conclusion, ADPKD patients have a more severely impaired maximal urine concentrating capacity with a lower maximal achieved urine urea concentration in comparison to IgAN control patients with a similar level of decreased kidney function. AVP secretion as response to water deprivation was similar in both groups. When disease progresses, AVP secretion upon thirsting increases in ADPKD patients. This increase in AVP can be harmful as AVP is known to enhance cell proliferation and cyst formation. Water deprivation may therefore be deleterious and should be avoided by ADPKD patients.

## Supporting Information

S1 File Dataset of all ADPKD patients and IgAN controls used to obtain the results displayed in this article. (XLSX)
